# A protocol for linking participants’ retailer ‘loyalty card’ records into the Avon Longitudinal Study of Parents and Children (ALSPAC)

**DOI:** 10.12688/wellcomeopenres.18900.1

**Published:** 2023-02-23

**Authors:** Anya Skatova, Andy Boyd

**Affiliations:** 1 MRC Integrative Epidemiology Unit, University of Bristol, Bristol, Avon, UK; 2 Population Health Sciences, Bristol Medical School, University of Bristol, Bristol, UK; 3 UK Longitudinal Linkage Collaboration, University of Bristol, Bristol, UK

**Keywords:** Longitudinal Population Studies, Digital Footprint, Shopping History Data, Loyalty cards, Data Linkage, Transactional Data, ALSPAC

## Abstract

Longitudinal population studies (LPS) have a long history of providing insights into how individual consumption patterns and other lifestyle choices affect health and socio-economic outcomes. LPS typically operate as research databanks, integrating rich and deep phenotypic data - covering diverse aspects of individual, family and household status - with genomic data and linked records on health and socio-economic outcomes. However, individual consumption and behavioural choices are traditionally studied solely using self-report methods which are prone to known biases. We propose to enrich LPS databanks with a new form of digital footprint data – individual shopping history records. These are collected by supermarkets through “loyalty” card schemes and can provide a new perspective on real world behaviours and history of consumption. However, as a novel class of data in the context of longitudinal research, our ability to assess the quality and completeness of the data is unknown, as is our ability to effectively triangulate between self-reported and linked data. This paper describes a protocol for linking individual level shopping history data into a LPS using Avon Longitudinal Study of Parents and Children (ALSPAC) as a testbed. The protocol covers the process of establishing participant fair processing, an ethical and legal basis for the linkage framework itself, and how these data will be integrated into the ALSPAC databank. It does not cover the subsequent research use of these data. The protocol was built on an extensive participant engagement and acceptability work and has been approved by the ALSPAC Law and Ethics committee.

## Introduction

Individuals’ interactions with companies, the Internet and digital services are now routinely recorded. These datasets provide a ‘digital footprint’ of online activities and transactions made through digital devices. Within this class of data, customer ‘loyalty card’ schemes are increasingly implemented by major retail companies as a mean to capture insights on their customers. Loyalty cards are also popular with consumers as they are incentivised through exclusive offers, discounts and other benefits. The records generated through loyalty cards are potentially an extremely useful source of data for longitudinal research. They provide granular, objective information on real world choices ( *e.g.*, in the domains of food, alcohol and tobacco consumption) and other behaviours ( *e.g.*, pain management, adherence to medication) directly related to pressing research topics. For this reason, the funders of UK longitudinal population studies (LPS) are now calling for studies to integrate transactional ‘digital footprint’ records into their data collection strategies ( DigitalFootprints, UKRI, 2022; ESRC Data Infrastructure Strategy, ESRC, 2022; Longitudinal Population Studies Strategy, Wellcome Trust, 2017).

Loyalty cards contain information about lifestyle choices and consumption patterns that are often resource- and time-consuming to obtain. Typically, LPSs have attempted to capture these data using self-report questionnaires or broadly similar modes capturing recalled/prospective information (such as fieldworker-conducted, or computer-assisted interviews). These measures are subject to well-described reporting and other biases ( Devaux & Sassi, 2016; Hebert *et al.*, 1995). Bias can be introduced as: firstly, such responses do not always accurately reflect behaviours due to issues such as social desirability and memory failures ( Hill & Davies 2001; Volk *et al.*, 2020); secondly, non-response to these assessments can be patterned by factors relating to the exposure/outcome of interest and may therefore introduce bias ( Rothman *et al.*, 2008) and thirdly, the temporal span of these measures ( *e.g.*, self-reported use of tobacco, food frequency diaries) cannot practically assess fluctuations in behaviours, seasonal differences or responses to life events. This suggests a potential to utilise objectively captured transaction data – such as store loyalty cards data - which are recorded at a high temporal frequency and are not subject to these biases; although, these may in turn be subject to different biases or sources of error.

The ability for digital footprint data, such as store loyalty cards, to accurately inform epidemiological or social science enquiry has raised concerns. These relate to the quality of the data, its coverage ( *i.e.*, missing transactions made in other retailers), potential omissions ( *e.g.*, where individuals’ make sensitive purchases without using a loyalty card), linkage error ( *e.g.*, where other individuals’ use the loyalty card) and population bias ( *e.g.*, where some population groups are less likely to shop at stores with loyalty card schemes). This is an under-developed area within longitudinal and other observational research due to historical difficulties in accessing these data and concerns regarding data protection legislation, privacy rights and acceptability amongst study participants. In order to realise full value of store transaction data for health research, it is necessary to link these data into LPS at an individual level to be able to investigate their biases and limitations.

LPSs, such as the Avon Longitudinal Study of Parents and Children (ALSPAC), are ideally placed to investigate these issues. They routinely collect a wealth of biomedical and social information about their participants, often over the course of decades and generations. A unique feature of these studies is the ability for direct participant involvement and engagement in the research design phase; which enables study staff and researchers to test the acceptability of new data collections, and to test feasibility with active participant involvement. This is underpinned by flexible data collection infrastructure necessary to support participant engagement, implement novel data collection mechanisms and to gain participant permission for the new use of their data. LPSs present an unrivalled opportunity for integrating digital footprint records, including store loyalty with self-reported data, wider linked health records (such as health records) and non-health government service interaction data ( *e.g.*, the provision of social security benefit records). Triangulating linked shopping records with self-reported data/wider linked records can serve as a ‘ground truth’ to validate patterns in transaction data and provide a testbed for identifying environmental exposures of risk factors for adverse health outcomes. Through extensive participant involvement, we have demonstrated that this use of participant records is considered broadly acceptable and likely to generate viable sample sizes ( Shiells *et al.*, 2020; Skatova *et al.*, 2019).

### Prior participant involvement and testing of acceptability

This protocol is informed by insights from ALSPAC participant engagement focus groups ( Skatova *et al.*, 2019), a wider participant involvement ( Shiells *et al.*, 2020), a questionnaire survey exercise conducted in 2018 (summary findings presented in Table 1) and an interview study conducted in 2021 (report on the interview results is available from the first author). The 2018 survey asked participants to provide information on their use of loyalty cards and the acceptability - in principle - of sharing these with ALSPAC. The survey suggests that of 4,462 respondents, 2,744 participants (65.4%) have at least one major UK supermarket or store loyalty card, and out of those 2,427 (88.4%) indicated that in principle they may be willing to share this data with ALSPAC.

**Table 1.** Results of a 2018 survey of ALSPAC participant held store loyalty cards and willingness to share.

	N (%) of people who hold cards by retailer (of 4,193 responding to the questionnaire)	N (%) of people who hold cards and expressed their willingness to share in the survey by retailer (out of 2,427 who indicated they are willing to share)
Boots Advantage Card	1,814 (43.26%)	1,350 (55.62%)
Tesco Clubcard	2,070 (49.38%)	1,532 (63.12%)
Sainsbury’s Nectar Card	1,654 (39.45%)	1,214 (50.02%)
Co-op Membership Card	526 (12.54%)	390 (16.07%)
Morrisons More Card	587 (14%)	438 (18.05%)
At least one of these five cards	2,744 (65.44%)	2,427 (100%)

For the 2021 interview study, 12 members of the ALSPAC index participants cohort were asked to download their loyalty cards data. The same participants then took part in a semi-structured telephone interview exploring their thoughts on the process of downloading the data, and their attitudes towards sharing this data in the future for linkage into the ALSPAC database. Whilst participants were broadly positive about the experience, they discussed various options for consent and suggested ideas for encouraging loyalty card donation. For example, interviewees suggested that a system for “labelling” projects by domain on the ALSPAC website could help participants decide which projects that use their data they want to opt out of. 

### Overview of the paper

**Figure 1.**
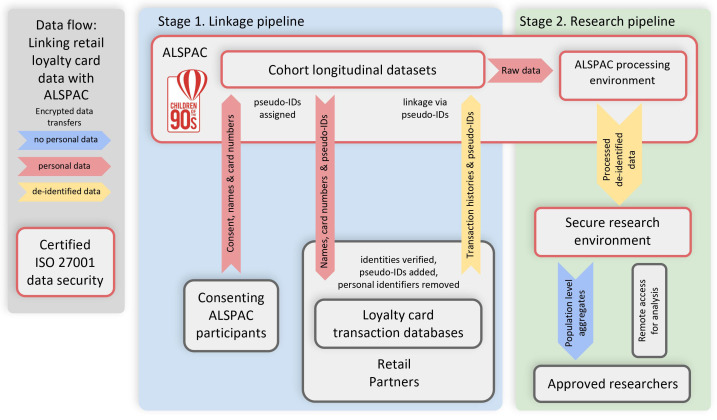
Loyalty Card data flow chart which summarises linkage and research pipeline procedure. Technical pilot described in this protocol will only focus on Stage 1/Linkage pipeline.

In this paper, we describe a protocol to consent the ALSPAC index participants for the research use of their store loyalty card data ( Figure 1). The protocol will be initially tested in a technical pilot and subsequently implemented in the full sample. The paper describes: (i) our objectives; (ii) the ALSPAC sample; (iii) the data contained in the loyalty cards; (iv) the technical pilot; (v) the full cohort linkage consent, linkage and data processing protocol; (v) participant involvement and research governance; (vi) outcomes; and (vii) data discovery and availability. This protocol forms part of a broader programme of ALSPAC scientific research which aims to build a rich and detailed research database: the use of which is intended to improve the public good through generating new understanding of complex health behaviours and outcomes.

## Methods

### Objectives

***Primary objective.*** Our primary objective is to enrich the ALSPAC database with new classes of participant behavioural data through linking individual-level shopping history records from major UK supermarkets and stores in a manner that is legally compliant and acceptable to both participants and the commercial companies who collect these records ( Figure 1). To do this we will seek participants’ explicit consent, develop reproducible data curation and analytical pipelines and promote the resulting data to the research community.

***Secondary objective.*** Our secondary objective is to provide an exemplar demonstration for the use of these novel data in a longitudinal context and establish a library of research tools ( *e.g.*, data curation and statistical syntax) that can aid wider LPSs and provide insights on the viability of digital footprint linkage strategies to the population data science community.

### Sample

ALSPAC is a multigenerational prospective birth cohort study. ALSPAC recruited pregnant women resident in and around the City of Bristol (South-West UK) and due to deliver between 1st April 1991 and 31st December 1992. There were an initial 14,541 enrolled pregnancies comprising 14,676 foetuses (for these at least one questionnaire has been returned or a “Children in Focus” clinic had been attended by 19/07/99). These pregnancies resulted in 14,062 live births and 13,988 children alive at one year. From age seven, attempts were made to recruit additional cases who were eligible under the original sample definition ( Boyd *et al.* 2013; Fraser *et al.* 2013). By age 24, an additional 913 index children had enrolled. The total sample size for analyses using any data collected after the age of seven is therefore 15,447 pregnancies, resulting in 15,658 foetuses. Of these, 14,901 were alive at one year of age ( Northstone *et al.*, 2019). The cohort has been followed intensively from birth through self-completed questionnaires and attending clinical assessment visits. ALSPAC has built a rich resource of phenotypic and genetic information relating to multiple genetic, epigenetic, biological, psychological, social, and other environmental exposures and outcomes. The ALSPAC website hosts a data dictionary that describes the available data.

***Inclusion and exclusion criteria.*** All enrolled ALSPAC index participants who have sole use/ownership ( *i.e.*, not a joint account) of a retailer loyalty card will be considered eligible for this study. Participants who have withdrawn from the study, have died or who are known to lack capacity to consent will be excluded. A subset of participants is lost to study contact, these individuals are considered eligible and will be included as new contact details are identified.

For the technical pilot we will recruit 10 participants from a random selection of participants who have previously reported active use of a loyalty card. For the full study we will invite all eligible participants including the pilot group.

### Loyalty cards data and coverage

Loyalty card records contain information about an individuals’ store transactions. The majority of major supermarket retailers in the UK operate loyalty cards schemes with high rates of population coverage. We surveyed ALSPAC participants about loyalty card utilisation in 2018 using a whole sample questionnaire ( Life@26+ questionnaire). This identified high levels of utilisation in ALSPAC participants ( Table 1) of the: Boots “Advantage Card”, Tesco “Clubcard”, Sainsbury’s “Nectar Card”, Co-op “Membership Card”, Morrisons “More Card”. Since 2018 a number of additional major retailers with high levels of market share have launched loyalty card schemes ( *e.g.*, Lidl Freebie Card and ASDA Rewards schemes). In the remainder of this paper we will refer to these stores as “the retailer”.

Store loyalty card data contains three categories of information – an illustrative example is provided in Table 2:

1) Information about the customer/card holder. This includes name and title, email address, home address, phone number(s), number of household members, date of birth, gender and various marketing preferences;
2) Summary records for each shopping visit: including, the total amount spent, amount saved on discounts, the exact time of the transaction, whether the purchase was online or in store, and if in store, address of the store and how the purchase was paid for ( *e.g.*, by card or cash).
3) Itemised information about each shopping trip purchase: this is very similar to the information presented on an itemised shopping receipt, including the names of items that were purchased, their quantity and price. For some supermarkets, each individual item has an assigned item code. This itemised information may include prescription and other ‘over the counter’ health-care products ( *e.g.*, pain relief medication).

**Table 2.** Transaction history recorded through loyalty cards.

Customer/card holder	
First Name	Joe
Surname	Bloggs
Email address	joe.bloggs@joebloggs.com
Phone number (day, evening, mobile)	07123123123
Title	Mr
Postal address	18 Hill Road, Hillville, Bristol, BS1 1BS
DOB	01.01.1991
Gender	Male
N of household members	1
Shopping basket * info	
Value of the basket	£14.45
In-store or online	In-store
Overall savings	£1.05
Store ID	1234
Store Address	Main Road, Hillville, Bristol
Store name	Store Hillville
Store format	Small Neighbourhood Store
Payment type	Visa Debit
Amount	£14.45
Time stamp	2021-01-04 10:32:38.530
**Product information**	
**Product 1**	
Name	Butchers 6 97% Pork Sausages 400G
Quantity	1
Price	£5
**Product 2**	
Name	Shiraz Wine 75Cl
Quantity	1
Price	£5.50
**Product 3**	
Name	Storebrand Rainbow Salad 320G
Quantity	2
Price	£2.50

*A basket includes products purchased at the same time

Store loyalty card data do not contain information about customers' financial details (such as bank or credit account details). Some information can be missing if the customer chooses not to provide it at the point of opening their card account ( *e.g.*, date of birth, address) and some customers may choose to provide false information.

### Technical pilot

A technical pilot is conducted to test the feasibility of the contact, consent and data linkage pipeline mechanisms (see ”Contact, consent and Data Linkage Protocol”). The pilot uses the same protocol as the full study. In addition to the main protocol, we ask the pilot participants to provide email feedback on the user experience. This feedback feeds into the iterative enhancement of the protocol ( *e.g.*, improvements to the clarity of instructions or the web-capture tool). The store loyalty card data collected through the pilot is destroyed after the evaluation of the pilot as the pilot only aims to test the technical feasibility of developed pipeline (with the pilot participants remaining eligible for the full study).

### Contact, consent and Data Linkage Protocol

The protocol describes the participant contact, consenting and provision of loyalty card information, the subsequent record linkage process and the extraction and transfer of copies of the records to the ALSPAC databank. A flow diagram summarising this process is shown in Figure 1. We will contact each retailer separately with data requests. The same protocol is applied to linking data from all the above-mentioned retailers.

***Contact and consent.*** Eligible participants will be selected from the ALSPAC administrative database and contacted by email (determined by participant preference and contact data availability). Participants will be sent an information sheet summarising the rationale for collecting and using loyalty card data in a longitudinal study, providing details of what data is requested, and how the data will be processed to safeguard participant rights. Participants will be asked to provide explicit (opt-in) consent and to provide details of their loyalty card(s) including the retailer details, account identification number and their personal identifiers (name, date of birth, address) as recorded on their loyalty cards account(s) - which could be a pseudonym. The consent includes permission for future, repeated, extracts of data unless the participant changes their consent status.

The ALSPAC website is being updated to include information reflecting the proposed linkage and data processing and information describing how to change consent status. ALSPAC provides ongoing fair processing information about this study via social media and print newsletters. All fair processing materials are designed using insights from our participant engagement activities and with input from ALSPAC’s panel of participant advisors (the ALSPAC Original Cohort Advisory Panel, OCAP) and ethical review committee (the ALSPAC Ethics and Law Committee, ALEC – A University of Bristol Faculty Ethics Committee) to ensure clarity and completeness.

The contact is managed and conducted by ALSPAC staff using the standard study mechanism. The consent and loyalty card information will be collected using a REDCap data management system ( Harris *et al.* 2009) which is used across ALSPAC data collection exercises and provides security and encryption data transfer. Participants have an option to upload a screenshot/photo of their loyalty card or type the details into the REDCap. Non-responders will be recontacted two weeks after the initial contact with a reminder email. All consent decisions will be uploaded from REDCap and stored and managed using the ALSPAC administrative database. ALSPAC staff will conduct validity and due diligence checks. In the event of a problem, ALSPAC staff will contact the participant by telephone or email. Participants are free to change their consent status at any time without impacting their wider involvement in the study.

For the technical pilot only, ALSPAC will contact participants by email after their REDCap response is received asking if the participants are willing to provide free-text feedback on their experience of participation in the study.

***Data Linkage Protocol.*** Project-specific pseudo-ID numbers will be assigned to each ALSPAC participant. For responding and consenting participants, the information from the REDCap system will be processed so that loyalty card ID numbers, participant names (the values provided on the loyalty card) and project pseudo-IDs are compiled into a linkage request file for each retailer. The files will be encrypted and transferred to the retailer. Decryption keys will be sent through a separate channel to maximise security.

The participating retailers use the provided information to identify participants records in their customer database and retrieve the matching transaction records for each participant. It is unlikely that retailers have capacity for probabilistic linkage methods and therefore a staged deterministic linkage protocol is implemented ( Table 3).

**Table 3.** Staged deterministic linkage protocol.

Linkage Stage	Identifiers	Criteria
1	store card ID number, username	Exact match on all identifiers
3	Forename, surname, date of birth, address *	Where store card ID is missing or invalid; where address is current or historical address.
4	First initial of forename, surname, date of birth, address *	Where store card ID is missing or invalid; where address is current or historical address.

* Where all identifiers are those provided by the participant as those used on their account details and for the purpose of this linkage

Where the data is linked, retailers compile a dataset of participant’s full retrospective record, excluding all direct participant identifiers. Each record is identified using the ALSPAC pseudo-ID. A match score is also provided ( *i.e.*, the deterministic algorithm which identified the link). These de-identified records will be encrypted and transmitted back to ALSPAC. For the technical pilot only, after the linkage is completed, participants shopping records and loyalty card numbers shared through consent form will be deleted from the ALSPAC database.

***Research analytical pipelines.*** Our objective is to produce two classes of data output: (1) rich transactional data which is de-identified yet retains maximum research utility and suited to deriving generalisable summary outcome variables; and (2) derived summary outcomes which have transformed row-level item data into research ready derived outcomes ( *e.g.*, the calorific value of a shopping basket).

Only ALSPAC data managers will process the raw, potentially identifiable, data. They will: (i) decrypt and store the data within the ALSPAC Data Linkage Safe Haven; (ii) create an immutable archive copy of the unprocessed data for disaster recovery purposes; (iii) convert the pseudo-ID to the ALSPAC ID used to integrate data across the ALSPAC database; and, (iv) conduct processing checks to ensure that all direct participant identifiers are removed or encrypted (including encrypting all store identifiers and location details; aggregating potentially disclosive information about store attributes). For further description of data environments as well as stakeholder roles and responsibilities ( *Extended data* [ Skatova & Boyd, 2023]).

**Figure 2.**
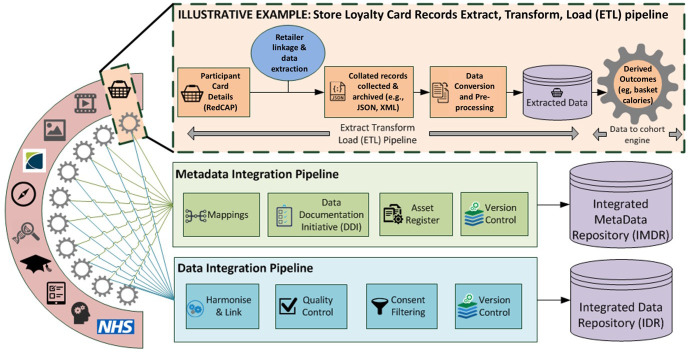
Store Loyalty Card ‘Extract, Transform, Load (ETL)’ data pipeline.

A data analytical pipeline will be established to enable algorithms to be developed to transform the full transactional record into derived outcomes ( Figure 2). ALSPAC Data Managers will de-identify the extracted records, ensuring identifiers such as store name and location are transformed into encrypted values and that the disclosure potential for indirect identifiers ( *e.g.*, regional product profiles) are assessed and controlled before release. Approved researchers are able to access the de-identified transactional data in a secure environment to build and validate data transformation algorithms ( *e.g.*, calculating the calorific value of baskets). These are applied by the ALSPAC data managers. The algorithm and accompanying documentation will be made available via the GitHub project.

After the data is linked, approved users will be able to conduct research using these data – linked with wider ALSPAC data including, where relevant, linked health, administrative and environmental records – within the Data Safe Haven.

***Prospective linkages.*** The linkage will be refreshed on a periodic basis. ALSPAC will provide a revised linkage file removing participants who have revoked consent or withdrawn from the study. The repeat linkages will use the same process as described above. Each update will include a full retrospective extract of data collected on each individual as the management of deltas (the difference between the current card records minus previously extracted card records) is likely to be too onerous across the range of suppliers.

These repeat linkages will provide a means to include additional participants: including those for whom the study have identified new contact details or those who have started to use loyalty cards.

### Research governance

***Participant involvement.*** This protocol has been developed through an extensive consultation with domain experts and ALSPAC participants. The research team has consulted the ALSPAC OCAP participant advisory group on four occasions at different stages of the project, has run focus groups with index participants ( Skatova *et al.*, 2019), consulted all index participants (see summary of responses in Table 1) and conducted a 2021 interview study assessing participants attitudes relating to linking shopping data into ALSPAC (for the description of results please contact the first author).

The consultations shaped this protocol through clarifying the range of data which are seen as acceptable to collect and those which are not (primarily, identifiable information about third parties, banking details such as account numbers); the need for rigorous de-identification in the data pipeline to provide confidentiality safeguards; and the design of clear participant fair processing materials which need to ensure the scientific utility of these linkages, which is explained as this is not intuitively understood by many participants. It is noted that many of the participants’ concerns are related to the wider safeguards of the ALSPAC databank ( *e.g.*, researcher approval process, transparency, the right to withdraw) and are out of scope of this protocol.

***Legal basis.*** This project will use identifiable participant information which constitutes personal data under Data Protection legislation and regulations. There is a possibility that individuals’ shopping transaction records will contain information that can be used to derive health status and is therefore considered to be potentially sensitive personal information and will be treated as such.

**Legal Gateway:** The University of Bristol’s Acts of Parliament provide a statutory basis for the University to conduct scientific research.

**Data Protection Legislation:** ALSPAC’s legal basis for using participant identifiable information, under UK GDPR and the Data Protection Act 2018, is: 1) performance of a task carried out in the public interest (Article 6(1)(e) in the GDPR); and, where sensitive personal information is involved, 2) scientific or historical research purposes or statistical purposes (Article 9(2)(j) in accordance with Article 89(1)).

**Common Law Duty of Confidentiality:** To address participant expectations around the confidentiality of their records, we provide fair processing materials setting a reasonable expectation as to how the study will use these data. This is evidenced through collecting explicit consent for data processing as well as pre-consent participant acceptability work.

**Participant rights**

- Right to Object: ALSPAC respects the right to withdraw and has a defined process for implementing this ( ALSPAC Research Ethics page). ALSPAC participants are able to select which aspects of the study they participate in without prejudicing involvement in other parts of the study ( *i.e.*, consenting or dissenting to this study is not a barrier to invitation or participation in other study activity).
- Right to Data Access: If participants wish to see their own data, they will be able to do so through exercising their right to data portability or subject access directly to the data holder ( *i.e.*, through contacting the retailer who will hold the most up-to-date and unfiltered version of the record). On participant request, ALSPAC will provide instructions how to request their loyalty cards data from retailers that were part of the linkage exercise.

***Ethical review.*** The protocol was approved by the ALSPAC Law and Ethics Committee - a dedicated and independently managed faculty ethics Committee as part of the University of Bristol Research Enterprise & Development function. Permission for the use of wider data collected via questionnaires, study ‘clinic assessment visits’ and linked routine records was obtained from participants following the recommendations of the ALSPAC Ethics and Law Committee at the time.

***Information security.*** The University of Bristol and ALSPAC, are UK GDPR-compliant. Many of the Information Security (IS) aspects are hosted or managed for ALSPAC by the University, however ALSPAC does have its own Senior Information Risk Officer (SIRO) and Standard Operating Procedures (SOP) which address risk registering and incident reporting. ALSPAC maintains a high level of IS training using external training providers, such as the Medical Research Council (MRC) and engages in regular internal audits. ALSPAC has been granted data security ISO 27001 certification. A Data Processing Impact Assessment (DPIA) has been completed and has been accepted by the University’s Data Protection Officer (DPO).

### Outcomes

The primary outcome of procedure described in this protocol is a linked shopping history dataset which is accessible for non-profit research intending to benefit the public good. The outcomes will include: (i) rates of participant consent broken down by the retailer; (ii) rates of valid linked data broken down by the retailer; (iii) the quantity and temporal coverage of records extracted. The descriptive statistics for these outcomes data will be made publicly available. Secondary outcomes include the usage and outcomes of these linked data by ALSPAC researchers, and the wider adoption of similar data linkage protocols by other longitudinal studies in the UK.

### Data discovery and availability

We will document these new data within an ALSPAC data dictionary entry and ‘Data Note’ publication. These will provide details on the study methods, results and descriptive analyses of the protocol outcomes (consent and linkage rates), linkage quality assessments, and will summarise the linked data. The documentation will be informed by the GUILD standard for reporting record linkage studies ( Gilbert *et al.*, 2018). The data will be added to the ALSPAC resource and made available under ALSPAC’s Data Access Policy. In line with ALSPAC’s wider data linkage policies, these data will only be available using one of ALSPAC’s secure analytical platforms ( *i.e.*, remote access to a secure data analysis system rather than a provision of data from ALSPAC to research institutions).

## Consent for publication

ALSPAC participants have been provided with fair processing materials describing the studies use of the data they have provided or those collected through record linkage and about the legal basis under which the study operates: this includes the sharing of de-identified data with researchers and the publishing of research findings. Study members have the right to withdraw from elements of the study or from the study entirely at any time. Full details of the ALSPAC consent/fair processing procedures are available from the study website ( https://www.bristol.ac.uk/alspac/participants/using-your-records/).

The study website also contains details of all the data that is available through a fully searchable data dictionary: http://www.bristol.ac.uk/alspac/researchers/data-access/data-dictionary/.

## Data Availability

ALSPAC data access is through a system of managed open access. The steps below highlight how to apply for access to the data referred to in this article and all other ALSPAC data. 1. Please read the ALSPAC access policy ( https://www.bristol.ac.uk/media-library/sites/alspac/documents/researchers/data-access/ALSPAC_Access_Policy.pdf) which describes the process of accessing the data and samples in detail, and outlines the costs associated with doing so. 2. You may also find it useful to browse our fully searchable research proposals database ( https://proposals.epi.bristol.ac.uk/), which lists all research projects that have been approved since April 2011. 3. Please submit your research proposal for consideration by the ALSPAC Executive Committee. You will receive a response within 10 working days to advise you whether your proposal has been approved. If you have any questions about accessing data, please email alspac-data@bristol.ac.uk. Open Science Framework: Skatova, A., & Boyd, A. (2023, February 22). Supplementary Materials for: A protocol for linking participants' retailer 'loyalty card' records into the Avon Longitudinal Study of Parents And Children (ALSPAC). https://doi.org/10.17605/OSF.IO/2ED6N ( Skatova & Boyd, 2023). Data are available under the terms of the Creative Commons Attribution 4.0 International license (CC-BY 4.0).
