# Supplementary material for: A protocol for linking participants’ retailer ‘loyalty card’ records into the Avon Longitudinal Study of Parents and Children (ALSPAC)
Source: Wellcome Open Res. 2024 Nov 8;8:99. Originally published 2023 Feb 23. [Version 2] doi: 10.12688/wellcomeopenres.18900.2 (PMC10474335; doi:10.12688/wellcomeopenres.18900.2)
Supplement: Peer review history

# Reviewer report: Reviewer response for version 2

**DOI:** 10.21956/wellcomeopenres.25723.r110700

**Published:** 2024-12-11

**Authors:** Laura Boeschoten

**Affiliations:** 1 Utrecht University, Utrecht, The Netherlands

Authors have appropriately responded to all my issues raised.

Is the study design appropriate for the research question?

Yes

Is the rationale for, and objectives of, the study clearly described?

Yes

Are sufficient details of the methods provided to allow replication by others?

Partly

Are the datasets clearly presented in a useable and accessible format?

Not applicable

Reviewer Expertise:

digital trace data, data donation, social media data, measurement error, survey methodology, missing data, data integration

I confirm that I have read this submission and believe that I have an appropriate level of expertise to confirm that it is of an acceptable scientific standard.

# Reviewer report: Reviewer response for version 2

**DOI:** 10.21956/wellcomeopenres.25723.r110701

**Published:** 2024-11-16

**Authors:** Jaakko Nevalainen

**Affiliations:** 1 Faculty of Social Sciences, Tampere University, Tampere, Finland

I am happy with the author responses to my concerns and have no further comments to make.

Is the study design appropriate for the research question?

Yes

Is the rationale for, and objectives of, the study clearly described?

Yes

Are sufficient details of the methods provided to allow replication by others?

Yes

Are the datasets clearly presented in a useable and accessible format?

Not applicable

Reviewer Expertise:

Data science, public health and nutrition

I confirm that I have read this submission and believe that I have an appropriate level of expertise to confirm that it is of an acceptable scientific standard.

# Reviewer report: Reviewer response for version 2

**DOI:** 10.21956/wellcomeopenres.25723.r110698

**Published:** 2024-11-13

**Authors:** Emily Lowthian

**Affiliations:** 1 Swansea University, Swansea, Wales, UK

Thank you for the reviews response, I appreciate their clarity. I am still concerned about the complexity for Figure 2, but if it cannot be changed then some information on how to read the figure would be helpful. 

 I look forward to seeing the results of the linkage, and follow-up studies.

Is the study design appropriate for the research question?

Yes

Is the rationale for, and objectives of, the study clearly described?

Yes

Are sufficient details of the methods provided to allow replication by others?

Partly

Are the datasets clearly presented in a useable and accessible format?

Yes

Reviewer Expertise:

Quantitative data research; longitudinal cohort studies; administrative data.

I confirm that I have read this submission and believe that I have an appropriate level of expertise to confirm that it is of an acceptable scientific standard.

# Reviewer report: Reviewer response for version 2

**DOI:** 10.21956/wellcomeopenres.25723.r110699

**Published:** 2024-11-12

**Authors:** James Goulding

**Affiliations:** 1 University of Nottingham, Nottingham, England, UK

I am happy with this version

Is the study design appropriate for the research question?

Yes

Is the rationale for, and objectives of, the study clearly described?

Yes

Are sufficient details of the methods provided to allow replication by others?

Yes

Are the datasets clearly presented in a useable and accessible format?

Not applicable

Reviewer Expertise:

Behavourial Science / Computer Science

I confirm that I have read this submission and believe that I have an appropriate level of expertise to confirm that it is of an acceptable scientific standard.

# Reviewer report: Reviewer response for version 1

**DOI:** 10.21956/wellcomeopenres.20957.r61583

**Published:** 2023-09-01

**Authors:** James Goulding

**Affiliations:** 1 University of Nottingham, Nottingham, England, UK

**Is the rationale for, and objectives of, the study clearly described? **This is a simple but potentially important piece of work, given  growing evidence that modelling of mass behavioural data such as shopping data (whether they are related to self-medication, food consumption or general activity partners) holds potential to shed light on a range of important social and health insights, from disease trajectories to syndromic surveillance.

 To ground such advances, however, much care and attention is required to ensure secure data collection, examination of representativeness, understanding of bias and to provide risk modelling of potential data leakages - key grounding blocks if the research community responsibly harness such data, and unpack the extent to which it can reflect the underlying conditions of donating individuals. As such the protocol described in this study appears a positive step within that research infrastructure given ts goals of investigating suitable data collection processes, storage, linkages and surrounding ethics ( and to examine whether insights gained they differ from traditional self-report methods).

 The proposal states these goals clearly, indicating its aims: to establish "participant fair processing" and "an ethical and legal basis for the linkage framework", as well as providing a clear methodology for "how these data will be integrated into the ALSPAC databank" (a specific national longitudinal cohort study, but one with similar characteristics to a broader set of cohorts). As such, I found the rationale and objectives clearly described.

**Is the study design appropriate for the research question? **Yes. The proposal's research question is centred around investigation of appropriate, secure, fair and ethical processes for ingestion and linkage of transactional data (within an existing longitudinal cohort) - and the simple study design reflects this appositely. The proposal also does not over-extend its scope, clearly stating that it will not explore the subsequent research use of such data. There does remain a Quonset here as to whether, in viewing final usage of ingested data as orthogonal to the task at hand, whether is possible to design an all encompassing process for data ingestion. Might different studies require different ingestion processes, anonymization and ethical requirements? Nonetheless, I took this question to be amongst these being addressed. 

 The proposed work additionally builds upon a 2021 pilot study, which obtained qualitative responses from a sample of existing ALSPAC participants, which adds to confidence in the researchers ability to implement their study design successfully, and answer such questions.

**Are sufficient details of the methods provided to allow replication by others? **The methods proposed in order to undertake this infrastructural investigation are pretty clear (with some useful accompanying figures provided as a useful addition). Overall transparency is aided by the fact that the process described by the authors is, in essence, also relatively simple (including the secure transmission from "loyalty card transaction databases" of data following consent from ALSPAC participants). However, I believe this simplicity is to the benefit of the study.

 The nature and coverage of the ALSPAC cohort, along with targeted participant sub-sample, are well set out. Inclusion and exclusion criteria from the study (e.g. loyalty card ownership) are stated, as is the nature and extent of PII ingested. Use of internal ALSPAC pseudo-IDs for data linkage is indicated, but I could not see information here of how they are generated (e.g. hashing approach). I assume also, however, as the study is leaning on existing ALSPAC processes, that this information is in other documentation.

 Finally, I noted that a two stage research pipeline described - one acting a raw transactional level (i.e. shopping line times) but with full de-identification of participants, and another at higher level of aggregated/feature engineering (such as summarization of basket calory counts). I was not 100% clear as to why only the first of these stage is explicitly list as "de-identified", nor how 'success' or 'failure' of each of these pipelines is to ascertained. Again, I suspect that being able to generate definitions concerning "success" or "failure" within a safe, research environment such as the ALSPAC cohort is part of the overall investigation. The fact that is clearly stated that the protocol was approved by the ALSPAC Law and Ethics Committee, went someway to providing confidence that this is indeed the case. 

 ----------------------

 Are the datasets clearly presented in a usable and accessible format?

 ----------------------

 Yes, in terms of the cohort being sampled - but most datasets have not yet been collected for the study (indeed this collection process is very much the body of the study itself).

Is the study design appropriate for the research question?

Yes

Is the rationale for, and objectives of, the study clearly described?

Yes

Are sufficient details of the methods provided to allow replication by others?

Yes

Are the datasets clearly presented in a useable and accessible format?

Not applicable

Reviewer Expertise:

Behavourial Science / Computer Science

I confirm that I have read this submission and believe that I have an appropriate level of expertise to confirm that it is of an acceptable scientific standard.

# Reviewer report: Reviewer response for version 1

**DOI:** 10.21956/wellcomeopenres.20957.r59636

**Published:** 2023-07-20

**Authors:** Laura Boeschoten

**Affiliations:** 1 Utrecht University, Utrecht, The Netherlands

This paper describes a protocol on how to obtain data from participant’s loyalty cards at various retailers and link these to data collected through a large cohort study. I’m particularly impressed by the magnitude of this study and by the fact that the retailers are interested in participating in this study. The majority of my comments relate to describing the processes of collaborating with the retailers in more detail. Particularly, you describe a protocol for ‘linking’ participants’ retailer ‘loyalty card’ records into the ALSPAC data. However, I wonder whether more work is/will be needed on integrating the data obtained through various retailers and would like to see either more detail on this in the linkage protocol, or a reflection on how the current work fits within a broader range of protocols where this may be described.

 In the abstract it is stated that “individual consumption and behavioral choices are traditionally studied solely using self-report methods which are prone to known biases” and in the introduction “they provide granular, objective information on real world choices”. Due to difficulty in accessing such digital trace data for research purposes, also much is still unknown about the quality of digital traces, so I would suggest to include a bit of a broader discussion on where the digital traces are expected to bring quality improvements to the study, and what elements are more to be investigated. In addition, you state that ‘triangulated linked shopping records with self-reported data/wider linked records can serve as a ground truth’, I think it would be good to elaborate a bit more on what you mean here exactly and how this ground truth is constructed.

 I find the opt-out and labelling suggestion from participants during the semi-structured interview very interesting and I’m curious to see how it will by implemented in the website.

 In table 1, I would also be interested to see a breakdown of how many loyalty cards of different retailers participants have and whether there are also participants who are only willing to share data from some retailers but not from all.

 At the section ‘Loyalty cards data and coverage’ you indicate that the loyalty card data contains three categories of information. Here, I am interested in whether this was the same for retailers, what instructions you provided to them in order to have the data in a similarly structured format for each retailer, whether there were discussions with retailers on what data they were for example not willing to provide, whether they all agreed, whether this was an iterative process, etc.

 Table 2 provides sections on customer/card holder, shopping basket info and product information. Here, I get the impression that I miss the information needed in order to link these three elements, like IDs, could you elaborate on that?

 At the part on ‘Data linkage protocol’ it is described that data is encrypted and transferred first to the retailer, and then back to ALSPAC. Can you elaborate on how this transfer took place? Did you provide the means and protocols for encryption as such that it was standardized between retailers?

 From the section ‘Research analytical pipelines’, you change in future tense. Did you perform all the steps until this moment? Maybe you can provide a provisional timeline of all the work that is planned and to what extent it has been carried out?

 Why is there no stage 2 in table 3?

 In the caption of figure 2, can you elaborate a bit more on how this figure should be read?

 At the ‘research governance’ section subsection ‘participant involvement’ you mention index participants. Can you elaborate on what you mean by this?

 At the section ‘Contact, consent, and data linkage protocol’ subsection ‘research analytical pipelines’ you describe two types of datasets that will be prepared with different intended users.

 At the section ‘Outcomes’, could you relate these intended outcomes more explicitly to these two planned datasets? Is the procedure described under ‘data availability’ only to access dataset 2 or also dataset 1?

Is the study design appropriate for the research question?

Yes

Is the rationale for, and objectives of, the study clearly described?

Yes

Are sufficient details of the methods provided to allow replication by others?

Partly

Are the datasets clearly presented in a useable and accessible format?

Not applicable

Reviewer Expertise:

digital trace data, data donation, social media data, measurement error, survey methodology, missing data, data integration

I confirm that I have read this submission and believe that I have an appropriate level of expertise to confirm that it is of an acceptable scientific standard, however I have significant reservations, as outlined above.

# Response

**Published:** 2024-10-23

**Authors:** Anya Skatova

**Affiliations:** University of Bristol, UK

This paper describes a protocol on how to obtain data from participant’s loyalty cards at various retailers and link these to data collected through a large cohort study. I’m particularly impressed by the magnitude of this study and by the fact that the retailers are interested in participating in this study. 

 I would suggest to include a bit of a broader discussion on where the digital traces are expected to bring quality improvements to the study, and what elements are more to be investigated. In addition, you state that ‘triangulated linked shopping records with self-reported data/wider linked records can serve as a ground truth’, I think it would be good to elaborate a bit more on what you mean here exactly and how this ground truth is constructed.

**Response: **This is a very interesting point but we suggest that it is out of scope of this protocol paper. The lead author published another paper recently that addresses some of these issues: Skatova A. Overcoming biases of individual level shopping history data in health research. NPJ Digital Medicine; 2024 https://www.nature.com/articles/s41746-024-01231-4. We also added this citation to the paper.

 I find the opt-out and labelling suggestion from participants during the semi-structured interview very interesting and I’m curious to see how it will by implemented in the website. **Response: **The opportunity to form different research projects is a process maintained by the data holder - in this case ALSPAC. All approved data linkage projects are published on ALSPAC website and participants have a grace period to write and ask to withdraw their data. 

 In table 1, I would also be interested to see a breakdown of how many loyalty cards of different retailers participants have and whether there are also participants who are only willing to share data from some retailers but not from all.

**Response: **We are planning to report this information in the follow up publication which is focusing on predictors of consent to link shopping data within LPS. We clarified this in the text of the paper. 

 At the section ‘Loyalty cards data and coverage’ you indicate that the loyalty card data contains three categories of information. Here, I am interested in whether this was the same for retailers, what instructions you provided to them in order to have the data in a similarly structured format for each retailer, whether there were discussions with retailers on what data they were for example not willing to provide, whether they all agreed, whether this was an iterative process, etc.

**Response: **All retailers are provided with the same request - to share what data they hold on consented participants. The structure of the data is broadly the same but will differ by retailer. We are still in the process of retrieving the data from retailers, and we will write up the description of the negotiations in the following publications. 

 Table 2 provides sections on customer/card holder, shopping basket info and product information. Here, I get the impression that I miss the information needed in order to link these three elements, like IDs, could you elaborate on that?

**Response: **The data is linked using loyalty card ID (which is indeed not in Table 2 because it depicts data under an “ID” or given account). In addition the linkage is checked against name and date of birth. 

 At the part on ‘Data linkage protocol’ it is described that data is encrypted and transferred first to the retailer, and then back to ALSPAC. Can you elaborate on how this transfer took place? Did you provide the means and protocols for encryption as such that it was standardized between retailers?

**Response: **The process is not standardised between retailers. 

 From the section ‘Research analytical pipelines’, you change in future tense. Did you perform all the steps until this moment? Maybe you can provide a provisional timeline of all the work that is planned and to what extent it has been carried out?

**Response: **This paper describes a data linkage protocol. It is hard to provide timelines because there are multiple complex stakeholders involved. But as we are going through the steps of completing our protocol and getting the data ready for research, we will be writing up all our findings and experiences. 

 Why is there no stage 2 in table 3?

**Response: **This is a typo and now is corrected. 

 In the caption of figure 2, can you elaborate a bit more on how this figure should be read? **Response: **We’ve added a couple of sentences clarifying the figure. 

 At the ‘research governance’ section subsection ‘participant involvement’ you mention index participants. Can you elaborate on what you mean by this?

**Response: **Index participants include “children” or the main ALSPAC cohort, who are around 30 years old. 

 At the section ‘Contact, consent, and data linkage protocol’ subsection ‘research analytical pipelines’ you describe two types of datasets that will be prepared with different intended users. At the section ‘Outcomes’, could you relate these intended outcomes more explicitly to these two planned datasets? Is the procedure described under ‘data availability’ only to access dataset 2 or also dataset 1?

**Response: **We added clarification to the Outcomes section.

# Reviewer report: Reviewer response for version 1

**DOI:** 10.21956/wellcomeopenres.20957.r61585

**Published:** 2023-07-20

**Authors:** Emily Lowthian

**Affiliations:** 1 Swansea University, Swansea, Wales, UK

This paper shows some promise towards safely and responsibly integrating loyalty data with existing participants in the ALSPAC study. Overall, I think the protocols and processes are well-positioned, and balance protecting the rights of participants while improving access to data for public good. I have a dozen or so comments about the paper and the protocol, but I think this is an excellent piece that has a wide range of positive outcomes if handled carefully. 

1. In the introduction, the authors discuss the 'concerns' around data protection - I think it may be worth outlining (a couple of sentences) what those concerns are and how they have developed over time?
2. I wonder when you discuss those with 'lacking' capacity, it may just be best described as without capacity? I wonder if 'lack' adds a nature of value to it. However, I'm not 100% on this so it might be worth checking to see what the general term is. 
3. It would be good to know the N of those who are ineligible for contact - it might be well described in a flow chart - but equally I appreciate this is not a cohort study.
4. Can the authors/data processes ensure DOB would not be accessible to the secondary researcher on the 'user' side? I'm not sure I see a rationale for use in secondary/research contexts. 
5. The false information will be difficult to navigate here if people are not transparent with information. What tests and checks will be put into place to authorise credible linkage?
6. Will the researchers ensure that the communication to share loyalty data was in simple, lay-friendly text so the participants could fully understand the research and implications/protections?
7. If participants change their consent, will their previous data be removed? Or if not, will there be an option clearly displayed to the participant if they wish to do so?
8. The decryption keys are an important process. It would be advantageous to know more information about this process; e.g. who will have access? How will they ensure no party has dual-access? Not only for participant reassurance, but for other longitudinal studies to replicate. Perhaps you are limited in providing information here via confidentiality which is understandable.
9. More information on deletion is needed - will this be permanent deletion? There are concerns with data having the ability to be restored via some technology back-ups, which risks participant confidence and trust. 
10. The algorithm sharing and accompanying documents will be a valuable resource.
11. I think Figure 2 could be improved to show the process of integrating loyalty data a little clearer; it is not clear on the outset, i.e. where to start reading. 
12. Are participants aware that if research is published which includes their data they cannot revoke their data use for that specific study? Or is that a possibility here?
13. I think the paper could discuss further outcomes in terms of the validity, reliability and general increase in robust research methods that surpass issues with self-reported measures. 
14. Final point, I think it would be worth stating the reviewer output processes that exist (i.e., non-disclosure checked by X reviewers, and the training required to access a TRE). This information improves the readers understanding of the security in which the data is held. 

Thanks for sharing such an interesting development.

Is the study design appropriate for the research question?

Yes

Is the rationale for, and objectives of, the study clearly described?

Yes

Are sufficient details of the methods provided to allow replication by others?

Partly

Are the datasets clearly presented in a useable and accessible format?

Yes

Reviewer Expertise:

Quantitative data research; longitudinal cohort studies; administrative data.

I confirm that I have read this submission and believe that I have an appropriate level of expertise to confirm that it is of an acceptable scientific standard, however I have significant reservations, as outlined above.

# Response

**Published:** 2024-10-23

**Authors:** Anya Skatova

**Affiliations:** University of Bristol, UK

This paper shows some promise towards safely and responsibly integrating loyalty data with existing participants in the ALSPAC study. Overall, I think the protocols and processes are well-positioned, and balance protecting the rights of participants while improving access to data for public good.

 In the introduction, the authors discuss the 'concerns' around data protection - I think it may be worth outlining (a couple of sentences) what those concerns are and how they have developed over time?

**Response: **We thank the reviewer for this comment but consider that outlining concerns around data protection a separate issue which is not the focus of the introduction for this protocol. To address reviewer’s comment, we included citations to two publications by us and colleagues that outline some of the concerns: Dolan, E.H., Shiells, K., Goulding, J. et al. Public attitudes towards sharing loyalty card data for academic health research: a qualitative study. BMC Med Ethics 23, 58 (2022). https://doi.org/10.1186/s12910-022-00795-8; and Skatova A, Shiells K and Boyd A. Attitudes towards transactional data donation and linkage in a longitudinal population study: evidence from the Avon Longitudinal Study of Parents and Children [version 2; peer review: 2 approved]. Wellcome Open Res 2021, 4:192 ( https://doi.org/10.12688/wellcomeopenres.15557.2). 

 I wonder when you discuss those with 'lacking' capacity, it may just be best described as without capacity? I wonder if 'lack' adds a nature of value to it. However, I'm not 100% on this so it might be worth checking to see what the general term is. 

**Response: **This is a standard phrase that is used in all ALSPAC publications to describe inclusion and exclusion criteria. 

 It would be good to know the N of those who are ineligible for contact - it might be well described in a flow chart - but equally I appreciate this is not a cohort study.

**Response: **For the purpose of this protocol, we have not contacted participants for consent. The following publications that will describe consent process will document Ns of participants who were contacted and who were not contacted and document the reasons. 

 Can the authors/data processes ensure DOB would not be accessible to the secondary researcher on the 'user' side? I'm not sure I see a rationale for use in secondary/research contexts. 

**Response: **Date of birth will not be accessible to secondary researchers (and neither will be any other identifiable information such as name). Note that table 2 reflects information that supermarkets collect together with loyalty cards data, not the data that will be made available for research. 

 The false information will be difficult to navigate here if people are not transparent with information. What tests and checks will be put into place to authorise credible linkage? **Response: **In case of linkage, if a participant includes false information (e.g., a made up loyalty card number or a different name to what is recorded in ALSPAC and supermarket database) we will not be able to complete the linkage for this person. As the data won't be linked, there are no checks to be done.  

 Will the researchers ensure that the communication to share loyalty data was in simple, lay-friendly text so the participants could fully understand the research and implications/protections?

**Response: **Participants’ communications is very important for ALSPAC (and indeed for other studies) and is produced together with ALSPAC participant communication and engagement group, with the input from ALSPAC Law and Ethics Committee, and participant engagement group. 

 If participants change their consent, will their previous data be removed? Or if not, will there be an option clearly displayed to the participant if they wish to do so?

**Response: **Participants can choose to withdraw their data before it is anonymised for analysis. In this case their data will be removed and not included in any further analysis. This is true for any ALSPAC data and clearly communicated on the study's website.  

 The decryption keys are an important process. It would be advantageous to know more information about this process; e.g. who will have access? How will they ensure no party has dual-access? Not only for participant reassurance, but for other longitudinal studies to replicate. Perhaps you are limited in providing information here via confidentiality which is understandable.

**Response: **Encryption and decryption will be performed by ALSPAC data manager. A counterpart in the supermarket will also have access to it in order to complete the linkage. After the data is linked all keys will be deleted. 

 More information on deletion is needed - will this be permanent deletion? There are concerns with data having the ability to be restored via some technology back-ups, which risks participant confidence and trust. 

**Response: **ALSPAC treats its data as any data holder and complies to UK GDPR laws. The data will be deleted if requested by a participant without ability for it to be restored. 

 The algorithm sharing and accompanying documents will be a valuable resource. **Response: **This is work in progress and we have not yet developed an algorithm for sharing and accompanying documentation. This will be published in due course. 

 I think Figure 2 could be improved to show the process of integrating loyalty data a little clearer; it is not clear on the outset, i.e. where to start reading. 

**Response: **Figure 2 needs to be read from left to write, however it depicts a complex process which is preserved on the figure. 

 Are participants aware that if research is published which includes their data they cannot revoke their data use for that specific study? Or is that a possibility here?

**Response: **This is a standard clause for any research - after the data is anonymised and submitted to analysis, and written up for publication, participants cannot retract their data from the dataset. This is clear from any research communication including ALSPAC website. 

 I think the paper could discuss further outcomes in terms of the validity, reliability and general increase in robust research methods that surpass issues with self-reported measures. **Response: **This is a very interesting point but we suggest that it is out of scope of this protocol paper. The lead author published another paper recently that addresses some of these issues: Skatova A. Overcoming biases of individual level shopping history data in health research. NPJ Digital Medicine; 2024: https://www.nature.com/articles/s41746-024-01231-4

 Final point, I think it would be worth stating the reviewer output processes that exist (i.e., non-disclosure checked by X reviewers, and the training required to access a TRE). This information improves the readers understanding of the security in which the data is held. **Response: **This is a great point, but again we suggest that it is out of scope for this protocol, which is focusing on linking data. We are working out the details of sharing outputs with shopping data and will publish the framework in due course.

# Reviewer report: Reviewer response for version 1

**DOI:** 10.21956/wellcomeopenres.20957.r55307

**Published:** 2023-04-05

**Authors:** Jaakko Nevalainen

**Affiliations:** 1 Faculty of Social Sciences, Tampere University, Tampere, Finland

This is a paper carefully describing the protocol for obtaining and linking retailer's loyalty card data to  well established cohort of parents and children. I feel that the task the authors will pursue is very ambitious and I will be very impressed if they succeed as planned, or even nearly so. The paper is very clearly written and an enjoyable read. I have only some minor comments to the authors:

**Introduction:**

 I am not really sure if loyalty card records provide information on adherence to medication to any meaningful extent. You can only observe what was purchased, not the doses actually taken, I think. The same confusion partially applies to pain management. Could the authors explain their argument?

 The last paragraph, triangulation. Why would this result to a ground truth? Records may indicate purchases to other people, for example. Therefore, they do not directly measure individual or even household consumption.

**Table 1.** n=4,193 did not match the respondent number in the text.

 I feel that some more information is needed on the retailers that are actually participating in this exercise. I did not spot if it was listed explicitly which ones are the ones that will be approached. (I suppose their final commitment remains as a risk). Could the authors provide their market shares to the reader? This would help an international reader to get a better idea of the retailer landscape.

**Methods**

 ALSPAC cohort is very well described including the time frames. But I did not find information on the time windows for retailers' data. What time periods will this cover?

 Are index participants both the mothers and their children, now in their thirties?

**Figure 1**: What exactly are the personal identifiers that are being removed before data transfer back to ALSPAC? For example, times of purchase or household size might be indirect ways to identify people in smaller areas.

**Categories of information:**

 How do the retailers maintain up-to-date information on size of the households? I don't understand that.

 Itemized information: Can the authors explain if the retailers have some common standards in registering the purchases? For example, are the purchased items always listed with the same names + volumes? Can they be easily grouped to nutritionally meaningful categories?

**Contact, consent** etc

 Participants will be contacted by email. To my experience, this might result into poor response rates. Will the authors have any information to which extent the lack of response was due to choice of nonparticipation, or simply missing the email (junk, invalid email addresses, etc?)

**Data linkage protocol.** Who holds the key for participant identity and pseudo ids, and who gets access to it?

 Perhaps a comment on indirect identifiers would be in order here as well.

**Prospective linkage.** It was mentioned that the retailers will provide entire new sets of data periodically to avoid difficulties with "delta". Many retailers (maybe not in UK) are legally forced to delete historical data after a certain period. How will this issue be dealt with?

Is the study design appropriate for the research question?

Yes

Is the rationale for, and objectives of, the study clearly described?

Yes

Are sufficient details of the methods provided to allow replication by others?

Yes

Are the datasets clearly presented in a useable and accessible format?

Not applicable

Reviewer Expertise:

Data science, public health and nutrition

I confirm that I have read this submission and believe that I have an appropriate level of expertise to confirm that it is of an acceptable scientific standard, however I have significant reservations, as outlined above.

# Response

**Published:** 2024-10-25

**Authors:** Anya Skatova

**Affiliations:** University of Bristol, UK

This is a paper carefully describing the protocol for obtaining and linking retailer's loyalty card data to well established cohort of parents and children. I feel that the task the authors will pursue is very ambitious and I will be very impressed if they succeed as planned, or even nearly so. The paper is very clearly written and an enjoyable read.

 I am not really sure if loyalty card records provide information on adherence to medication to any meaningful extent. You can only observe what was purchased, not the doses actually taken, I think. The same confusion partially applies to pain management. Could the authors explain their argument?

**Response: **We agree with the reviewer that on its own loyalty card records do not provide information about adherence to medication. However, they would provide this information if combined with medical records, as one can study discrepancy of what has been prescribed vs purchased. This still leaves the purchase - consumption gap opened which is true to any conclusions derived from purchasing data. In addition, in some cases - e.g. repeated purchases - we can reasonably assume that medications were consumed.  In terms of pain management - purchases of pain medication such as ibuprofen or paracetamol provide indication of individual managing pain. Again, through looking at patterns of repeated purchases we can reasonably assume that medication was consumed but purchase - consumption gap still remains a barrier of using shopping data for health research.  To tone down the claim, we inserted “in relation to” before pain management, adherence to medication in the first para of the introduction.  

 The last paragraph, triangulation. Why would this result to a ground truth? Records may indicate purchases to other people, for example. Therefore, they do not directly measure individual or even household consumption.

**Response: **In this case we refer to self-reported data/wider linked records  “ground truth” for shopping data. We hope this clarifies the confusion. 

 Table 1. n=4,193 did not match the respondent number in the text.

**Response: **Thank you for this comment. We checked all numbers in Table 1 and in prior text and corrected them. The numbers now deviate from the previous version although percentages are similar. This occurred due to error in the code and in addition some participant data being not incorporated into the database at the time of analysis for the initial draft of the paper. 

 I feel that some more information is needed on the retailers that are actually participating in this exercise. I did not spot if it was listed explicitly which ones are the ones that will be approached. (I suppose their final commitment remains as a risk). Could the authors provide their market shares to the reader? This would help an international reader to get a better idea of the retailer landscape.

**Response: **At the point of writing this protocol paper, we have not made any formal arrangements with listed supermarkets and it is correct that their final commitment remains a risk. However, since the protocol has been published, we have already been able to retrieve one of the provided data - namely Sainsbury’s - with the rest to follow shortly. We will be publishing a separate piece to summarise linked data.  In terms of market share, based on Statistica ( https://www.statista.com/statistics/280208/grocery-market-share-in-the-united-kingdom-uk/) in April 2024 Tesco took 27.4%, Sainsbury’s 15.3%, Co-op 5.4% and Morrisons 8.7%. Boots is a beauty retailer so it is hard to directly compare. We have not put this info into the paper as the protocol is not reporting linked data which will be subject to a separate publication.  

 Methods

 ALSPAC cohort is very well described including the time frames. But I did not find information on the time windows for retailers' data. What time periods will this cover?

**Response: **The data provided by retailers is bound by the timeframes for which they store data. In our experience that varies between different providers, and can be anything between 2 last year to 10 or more. 

 Are index participants both the mothers and their children, now in their thirties?

**Response: **Index participants only include “children” who are now in their 30's. 

 Figure 1: What exactly are the personal identifiers that are being removed before data transfer back to ALSPAC? For example, times of purchase or household size might be indirect ways to identify people in smaller areas.

**Response: **In this case we specifically mean direct identifiers such as names. We agree with the reviewer that other information can be used to identify individuals in smaller areas, however at this point the data is not shared with researchers since only ALSPAC data managers have access to the ALSPAC processing environment. This is the point when disclosure checks on raw data are conducted to ensure that no information that can potentially lead to identify a participant.  

 Categories of information: How do the retailers maintain up-to-date information on the size of the households? I don't understand that.

**Response: **Some retailers ask for this information at the point when an individual subscribes to a loyalty card scheme. In addition, retailers often categorise individuals into different groups, and infer whether the shopping on the card belongs to an individual, a family as well as note family composition. The latter could not be accurate. 

 Itemized information: Can the authors explain if the retailers have some common standards in registering the purchases? For example, are the purchased items always listed with the same names + volumes? Can they be easily grouped to nutritionally meaningful categories? In the UK retailers do not have common standards in registering the purchases. 

 Contact, consent etc. Participants will be contacted by email. To my experience, this might result into poor response rates. Will the authors have any information to which extent the lack of response was due to choice of nonparticipation, or simply missing the email (junk, invalid email addresses, etc?)

**Response: **This is a standard way that ALSPAC is contacting participants. While it would be an excellent idea to estimate reasons for no response, unfortunately we don’t have information due the factors described above. 

 Data linkage protocol. Who holds the key for participant identity and pseudo ids, and who gets access to it? ALSPAC data manager holds key information with regards to participant identity and matches to pseudo IDs.

**Response: **This information is shared with the supermarket for the purpose of data linkage. Note that supermarkets already have these people’s names, the only new piece of information that is shared is that they are participants in the study. 

 Perhaps a comment on indirect identifiers would be in order here as well.

**Response: **We are not quite sure which indirect identifiers the reviewer means. 

 Prospective linkage. It was mentioned that the retailers will provide entire new sets of data periodically to avoid difficulties with "delta". Many retailers (maybe not in UK) are legally forced to delete historical data after a certain period. How will this issue be dealt with?

 ​​​​​​​ **Response: **This is a new project and way of data collection. We are aware that not all supermarkets might have data for similar time periods and we will record these discrepancies in our reports. Note that “updates” will refer to new data collected in the past year or two, and this is the data that is most likely to be still stored by the supermarket.
